# Executive function in children with disruptive mood dysregulation disorder compared to attention-deficit/hyperactivity disorder and oppositional defiant disorder, and in children with different irritability levels

**DOI:** 10.1007/s00787-023-02143-6

**Published:** 2023-01-21

**Authors:** Astrid Brænden, Marit Coldevin, Pål Zeiner, Jan Stubberud, Annika Melinder

**Affiliations:** 1 Division of Mental Health and Addiction, Oslo University Hospital, Sognsveien 22, 0372 Oslo, Norway; 2 Lovisenberg Diaconal Hospital, Nic Waals Institute, Oslo, Norway; 3 Institute of Clinical Medicine, University of Oslo, Oslo, Norway; 4 Department of Research, Lovisenberg Diaconal Hospital, Oslo, Norway; 5 Department of Psychology, University of Oslo, Oslo, Norway

**Keywords:** Disruptive mood dysregulation disorder, Executive function, Irritability, Attention-deficit/hyperactivity disorder, Oppositional defiant disorder

## Abstract

Addressing current challenges in research on disruptive mood dysregulation disorder (DMDD), this study aims to compare executive function in children with DMDD, children with attention-deficit/hyperactivity disorder (ADHD), and children with oppositional defiant disorder (ODD). We also explore associations between irritability, a key DMDD characteristic, and executive function in a clinical sample regardless of diagnosis. Our sample include children (6–12 years) referred to child psychiatric clinics. Measures of daily-life (parent-reported questionnaire) and performance-based (neuropsychological tasks) executive function were applied. Identifying diagnoses, clinicians administered a standardized semi-structured diagnostic interview with parents. Irritability was assessed by parent-report. First, we compared executive function in DMDD (without ADHD/ODD), ADHD (without DMDD/ODD), ODD (without DMDD/ADHD) and DMDD + ADHD (without ODD). Second, we analyzed associations between executive function and irritability using the total sample. In daily life, children with DMDD showed clinically elevated and significantly worse emotion control scores compared to children with ADHD, and clinically elevated scores on cognitive flexibility compared to norm scores. Children with DMDD had significantly less working memory problems than those with ADHD. No differences were found between DMDD and ODD. Increased irritability was positively associated with emotional dyscontrol and cognitive inflexibility. For performance-based executive function, no diagnostic differences or associations with irritability were observed. We discuss how, in daily life, children with high irritability-levels get overwhelmed by feelings without accompanying regulatory capacities.

**Supplementary Information:**

The online version contains supplementary material available at 10.1007/s00787-023-02143-6.

## Introduction

Irritability is an unpleasant feeling defined by a low threshold for experiencing and demonstrating anger [1]. Even though irritability is a frequent mood state, increased and prolonged levels of irritability in children may be symptomatic of disruptive mood dysregulation disorder (DMDD). DMDD is defined by chronic irritability (most days in ≥ 1 year) and frequent temper outbursts (≥ 3 per week), present in two or more settings [2]. Irritability can also be a symptom of other psychological disorders, but DMDD is the only diagnosis where irritability is a core feature and a required diagnostic criterion. The concept of irritability has been widely neglected in psychopathology research, but has in recent years become an increased focus of research (cf. DSM-5’s inclusion of DMDD in 2013). Irritability is among the most common reasons for which families seek child mental health services [3]. Irritability is linked to severe outcomes such as high levels of functional impairments, poor psychological and physical health [4, 5], suicidality [6], and continued impairment in adulthood [7].

In child mental health services, DMDD, oppositional defiant disorder (ODD), and attention-deficit/hyperactivity disorder (ADHD) are among the most common disorders [8, 9] and are all related to irritability. Like DMDD, ODD is characterized by irritable mood, as well as argumentative behavior, or vindictiveness [2]. Thus, children with ODD may present with irritability, but irritability is not a required symptom. In DSM-5, DMDD and ODD are considered an affective versus a conduct disorder, respectively [2]. In contrast, clinical irritability is considered a subtype of ODD with chronic irritability-anger in the ICD-11 [10]. Accordingly, there is a need to investigate if there are different mechanisms between DMDD and ODD to gain clarity in how clinical irritability should be understood and applied in diagnostic manuals.

ADHD is defined by inattention, hyperactivity, and impulsivity [2]. Evidently, irritability is not a criterion for ADHD. Recent research reveals, however, that children with DMDD often fulfills the criteria for ADHD [11–13]. Importantly, even though research shows that most children with DMDD have ADHD, this is not true for all children with DMDD. A new systematic review hypothesizes that children with DMDD primarily have difficulties in executive functions (EF) related to emotion regulation, whereas children with ADHD struggle with EF more broadly, i.e., also in less emotionally aroused situations [14].

Emerging evidence suggests that higher levels of irritability in childhood is linked to executive dysfunction [15–18]. EF is a set of cognitive processes that enable deliberate, top-down regulation of thought, behavior, and emotion [19, 20]. EF develops gradually during childhood [21, 22] and plays a central role in the etiology of psychopathology [23]. Importantly, maladaptive levels of irritability may reflect failure to engage neuropsychological skills necessary for effective emotion regulation [16]. In children, EFs can be measured using NEuroPSYcological (NEPSY-2) [24] tasks in standardized, relatively emotionally neutral and highly structured environments with directions from an examiner. For more ecological measures of children’s EFs, parents can provide daily-life information using the Behavior Rating Inventory of Executive Function (BRIEF-2) [25].

Despite its clinical significance, only one study has examined EF in children with DMDD specifically [26]. Unfortunately, this study has clear methodological limitations such as unclarity regarding multiple comparison corrections and the diagnostic assessment method. DMDD originates from severe mood dysregulation (SMD; [27]) which was developed to test if non-episodic irritability is a child version of bipolar disorder (which it was not; [5, 7, 28]) after the rates of bipolar disorder in children had increased substantially [29]. Due to the scarcity of studies examining EF in DMDD, research using SMD criteria [30] could give insights to EF in DMDD. However, the results from SMD research concerning EF are inconsistent and ambiguous [11, 15, 31–33]. A considerable problem in current SMD and DMDD research is the high ADHD comorbidity, making it difficult to identify specific DMDD mechanisms [14].

Beneficial and complementary insights to the EF in children with different irritability-levels can be achieved using dimensional approaches to irritability. Currently, results from such studies indicate either a weak or no relationship between irritability and performance-based EF but that irritability is linked to neural activations in brain areas associated with EF [16–18]. These studies have not, however, examined the relationship between irritability and perceived EF in daily life.

### The present study

Here, in a sample of 6–12-year-old children referred to two child psychiatric units, a diagnostic and a dimensional approach is used to examine irritability and EF by neuropsychological tasks and parent-report. We first compare EFs in children diagnosed with DMDD without ADHD comorbidity from children with ADHD-only and compare those groups to children with DMDD + ADHD, as well as to children with ODD-only. Second, regardless of diagnostic groups, we explore the associations between irritability and EF in the total sample.

We expect that (1) children with DMDD will show clinically elevated and significantly worse scores on emotion control by parent-report compared to children with ADHD, and (2) higher levels of irritability to correlate significantly with poor emotion control by parent-report. We made no further hypotheses due to the limited knowledge on the relationship between EF, irritability, and DMDD.

## Methods

### Participants

**Fig. 1:**
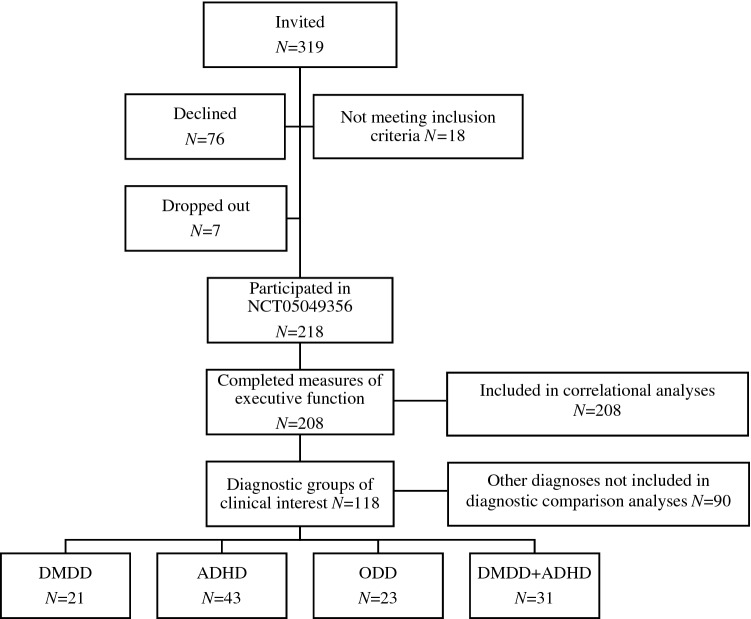
Selection process

The present study included a transdiagnostic treatment-seeking sample of 208 children (see Table 1). All participants were included in the analyses exploring the associations between irritability and executive function, whereas 118 of these fulfilled the diagnostic criteria for DMDD (without ADHD/ODD), ADHD (without DMDD/ODD), ODD (without DMDD/ADHD) or DMDD + ADHD (without ODD) and were included in the comparison analyses. Participants were recruited among primary school attendants (in Norway, 6–12 years) referred to outpatient clinics of child psychiatry at Oslo University Hospital and Nic Waals Institute between January 2019 and August 2021. That is, these children had not received psychiatric treatment or medication at the time of inclusion and study participation. Informed consent was attained from parents. The study was approved by the Regional Committees for Medical and Health Research Ethics (#2017/135) and is part of a registered study protocol (NCT05049356). Inclusion criteria included children between 6 and 12 years of age, IQ ≥ 70, and Norwegian language-skills good enough to respond to questionnaire and semi-structured clinical interview. The selection process is presented in Fig. 1 (see Supporting Information I for a description of the effects of COVID-19 on data collection).

**Table 1:** Participant characteristics

	Total sample *n* = 208	DMDD *n* = 21	ADHD *n* = 43	ODD *n* = 23	DMDD + ADHD *n* = 31	Group comparison^a^
Age *M* (SD)	9.7 (1.8)	9.2 (1.7)	9.6 (1.8)	9.9 (1.5)	9.4 (2.0)	*F*(3,114) = 0.44, *p* = 0.73
Girls *N* (%)	82 (39)	6 (27)	16 (37)	10 (42)	6 (19)	*χ*^2^ (3,118) = 4.30, *p* = 0.23
FSIQ *M* (SD) Range 66–152	99.4 (15.5) *n* = 142	100.1 (15.8) *n* = 13	100.0 (18.8) *n* = 33	98.2 (8.4) *n* = 15	98.1 (11.7) *n* = 24	*F*(3,80) = 0.22, *p* = 0.88
GAI M (SD) Range 84–94	90.0 (5.3) *n* = 3					
Family income^b^ *N* (%)						*χ*^2^ (3, 100) = 4.37, *p* = 0.23
< 700.000 NOK	50 (28)	6 (33)	14 (38)	3 (14)	10 (38)	
> 700.000 NOK	129 (72)	12 (66)	23 (62)	19 (86)	16 (62)	
Parental work%^c^ *M* (SD) Range 0–200	156.8 (61.9)	145 (74)	157 (55)	164 (58)	140 (78)	*F*(3,97) = 0.53, *p* = 0.66
Living situation *N* (%)						*χ*^2^ (3,116) = 5.45, *p* = 0.14
Common^d^	155 (76)	15 (71)	29 (69)	22 (92)	20 (65)	
Shared^d^	50 (24)	6 (29)	13 (31)	2 (8)	11 (35)	
Diagnoses/comorbidity *N* (%)						
DMDD	52 (25)	21 (100)	–	–	31 (100)	
ADHD	87 (42)	–	43 (100)	–	31 (100)	
ODD	36 (17)	–	–	23 (100)	–	
Anxiety	126 (61)	10 (45)	30 (70)	19 (83)	17 (55)	
Depression	23 (11)	4 (19)	3 (7)	3 (13)	3 (10)	
Conduct disorder	10 (5)	3 (14)	1 (2)	–	2 (7)	
Trauma	8 (4)	–	–	–	2 (7)	
Tics	14 (7)	–	1 (2)	2 (9)	3 (10)	
No comorbidity	88 (33)	8 (38)	11 (26)	4 (17)	11 (36)	

*ADHD Diagnoses* = combined type, inattentive type, hyperactivity/impulsivity type and ADHD not otherwise specified. Conduct disorders = conduct disorder and conduct disorder not-otherwise-specified. Anxiety = anxiety disorders; social anxiety, separation anxiety, generalized anxiety disorder, panic disorder and phobias. Depressive disorders (DMDD excluded) = depressive episodes, dysthymia and depression not-otherwise-specified. Trauma = post-traumatic stress disorder and acute stress disorder. Tic disorders = Tourette’s syndrome, persistent motor and/or vocal tics, and provisional motor and/or vocal tics

*FSIQ* Full Scale IQ, *GAI* General Ability Index

^a^Comparing DMDD, ADHD, ODD, DMDD + ADHD

^b^Family income per year where the child lives

^c^Range: 0% (none of the parents are working) to 200% (both parents working full-time)

^d^Living situation: Common = the child lives full-time with both parents. Shared = the child lives part-time with each parent

### Measures

Research measures were included as part of each patient’s standard clinical assessment and took place after referral and before potentially further assessment or treatment.

*Schedule for Affective Disorders and Schizophrenia for School-Age Children Present and Lifetime Version (K-SADS-PL-5)* [34] was used to determine the psychiatric diagnoses. K-SADS-PL-5 is a validated semi-structured diagnostic interview corresponding with DSM-5 diagnoses frequently applied in research and clinical practice [35]. Thirteen clinical psychologists and final year clinical psychology students administered the Norwegian version of K-SADS-PL-5 with parents. Reliability was established in three ways: first, interviewers were trained in the administration of K-SADS-PL-5 before they contributed to the data collection. Secondly, cases were discussed in conference with other clinicians. Thirdly, 9% (*n* = 19) of the interviews were scored independently by two clinicians, demonstrating a substantial agreement between the interviewers’ diagnostic evaluations (Cohen’s к = 0.80).

*Child Behavior Check List (CBCL)* [36], was completed by parents. Irritability was measured using the CBCL Irritability scale that consists of three CBCL items: (I) temper tantrums or hot temper, (II) stubborn, sullen, or irritable, and (III) sudden changes in mood or feelings. Items are rated on a 3-points Likert-scale (not true, sometimes true, often true) with a range of 0 (none) to 6 (high). Raw scores were used in the statistical analyses.

*Behavior Rating Inventory of Executive Function 2nd edition (BRIEF-2*) [25] is designed to measure children’s EF in social and behavioral contexts. Parents rated the frequency of behavior on a 3-point Likert-scale (never, sometimes, often). The BRIEF-2 includes the following scales: Inhibit (also referred to as Inhibition), Self-Monitor, Shift (also referred to as Cognitive Flexibility), Emotion Control, Initiate, Working Memory, Plan/Organize, Task-Monitor, and Organization of Materials. Raw scores were converted to T-scores based on validated norms [25]. Raw scores were used in the statistical analyses and T-scores were used to examine whether the group’s scores were within normal or clinical range compared to standardized norm values. T-scores ≥ 65 is regarded as in the clinical range [25], indicative of clear problems. For participants with < 5% missing item response, the median value for all participants (*n* = 188) was imputed.

*NEPSY Second-edition (NEPSY-2)* [24] is a performance-based neuropsychological assessment tool. Participants completed three subtests: (1) Design Fluency (for children between 5 and 12 years) assessing behavioral productivity and the child’s ability to generate unique designs by connecting five dots, presented in a structured and random way, (2) Inhibition measuring the ability to inhibit automatic responses (5–16 years) and to switch between response styles (7–16 years), and (3) Word List Interference (7–16 years) assessing verbal working memory. Raw scores were transformed to standardized scaled scores by Pearson’s NEPSY-2 machine-based scoring-tool. The scaled scores were used in the statistical analyses, and to examine whether the group’s scores were within normal range compared to standardized norm values. Scaled scores < 8 are indicative of clinical scores, clearly below normal levels.

*Full Scale IQ (FSIQ) or General Ability Index (GAI)* was estimated using the Wechsler Intelligence Scale for Children, fifth edition [37] if the healthcare professional responsible for the child’s treatment found it necessary as part of the child’s clinical assessment.

### Statistical procedures

Analyses were done using R 1.3.1093 [38] and IBM SPSS Statistics Version 28.0.0.0 (190). Alpha was decided to 0.05. Characteristic differences between diagnostic groups were analyzed using one-way analysis-of-variance (ANOVAs). The effects of sex and age on the measured variables were also examined. ANOVAs or analysis-of-covariance were conducted to examine differences between diagnostic groups. Effect sizes using partial eta squared with 0.01, 0.06, and 0.14 were interpreted as small, medium, and large effects, respectively [39, 40]. Pairwise comparisons with Bonferroni adjustments were done to investigate statistically significant differences between groups. Pearson correlation analysis was used to test the association between irritability and EFs. Observed power (β) was calculated to investigate the probability of Type-II error (see Supporting Information II for sample size estimates).

## Results

Participant characteristics are presented in Table 1 showing no significant differences between the four diagnostic groups on demographic variables. Levene’s test showed that across diagnostic groups homogeneity of variance could be assumed. Sex differences were found on all BRIEF-2 raw scale scores, *p*’s < 0.05, except for emotion control, *p* = 0.15. No sex differences were found on any NEPSY-2 scales. Age did not correlate with any NEPSY-2 scales, but correlated significantly with BRIEF-2 inhibition, self-monitor, cognitive flexibility and emotion control, *r*’s between − 0.19 and − 0.36, *p*’s < 0.05,* N* = 189. Age and sex are therefore included in further analyses for these variables.

### Diagnostic groups × Executive function (parent-report)

**Fig. 2:**
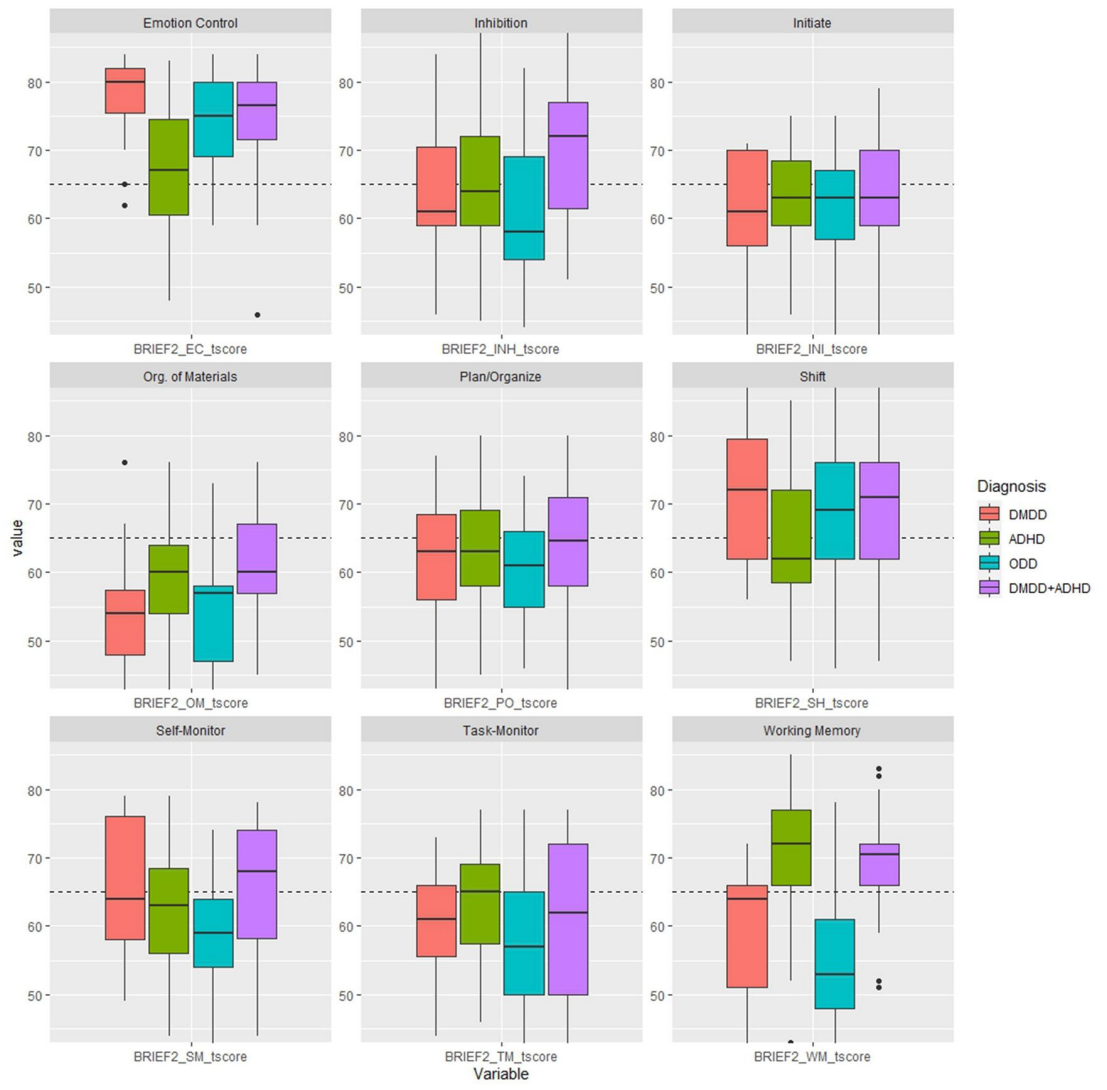
Diagnostic comparison and illustration of clinically elevated BRIEF-2 T-scores. Dashed line = clinical elevated score, i.e., T-Scores > 65

The results from the comparison analyses on BRIEF-2 are presented in Table 2. Significant differences were found in emotion control, inhibition, working memory, and organization of materials. All groups had clinically elevated scores on emotion control, as depicted in Fig. 2, but children with DMDD including those with DMDD + ADHD had significantly higher scores than children with ADHD. No significant differences were found between children with DMDD or ODD, but children with ODD had significantly higher scores on emotion control than those with ADHD-only. Compared to norm scores, children with DMDD showed clinically elevated scores on cognitive flexibility, but there were no significant differences between groups. Children with ADHD including those with DMDD + ADHD had clinically elevated scores and significantly higher scores on working memory than both children with DMDD or ODD.

**Table 2:** Executive function differences between diagnostic groups (*n* = 118)

*M* (SD)	DMDD	ADHD	ODD	DMDD + ADHD	Analysis of variance	*η*_p_^2^	*β*	Pairwise Comparison (Bonferroni adjusted)
BRIEF-2 (Raw scores)								
Emotion control^a^	21.7 (2.3)	17.7 (3.7)	20.9 (2.5)	20.5 (3.5)	*F*(3,100) = 8.49, *p* < 0.001	0.20	0.992	DMDD – ADHD*** ADHD – DMDD + ADHD** ADHD – ODD**
Inhibition^b^	17.3 (3.1)	17.3 (3.9)	15.5 (3.1)	19.4 (3.1)	*F*(3,99) = 4.87, *p* = 0.003	0.13	0.897	ODD – DMDD + ADHD**
Self-monitor^b^	9.4 (2.0)	8.8 (1.9)	7.9 (1.7)	9.3 (2.0)	*F*(3,99) = 2.38, *p* = 0.07	0.07	0.581	–
Cognitive flexibility^b^	18.0 (3.1)	15.9 (3.6)	17.1 (3.5)	17.5 (3.6)	*F*(3,99) = 1.79, *p* = 0.15	0.03	0.454	–
Initiate^c^	10.4 (2.0)	10.9 (1.8)	10.3 (1.9)	10.9 (2.3)	*F*(3,100) = 0.39, *p* = 0.76	0.01	0.124	–
Working memory^c^	16.1 (3.6)	20.1 (3.3)	14.4 (3.7)	19.4 (2.8)	*F*(3,100) = 16.8 *p* < 0.001	0.34	1.000	DMDD – ADHD*** DMDD – DMDD + ADHD** DMDD + ADHD – ODD*** ADHD – ODD***
Plan/organize^c^	17.5 (3.7)	18.2 (3.0)	16.8 (2.8)	18.3 (3.7)	*F*(3,100) = 0.84, *p* = 0.48	0.03	0.227	–
Task-monitor^c^	11.2 (2.6)	12.1 (2.3)	10.4 (2.9)	11.2 (3.2)	*F*(3,100) = 1.53, *p* = 0.21	0.04	0.392	–
Organization of materials^c^	10.9 (3.0)	12.5 (2.7)	11.0 (2.7)	13.1 (2.8)	*F*(3,100) = 3.70, *p* = 0.01	0.10	0.791	DMDD – DMDD + ADHD*
NEPSY-2 (Scaled scores)								
Cognitive flexibility	9.0 (3.5)	8.6 (3.0)	8.0 (2.8)	8.3 (3.1)	*F*(3,101) = 0.36, *p* = 0.78	0.01	0.119	–
Inhibition-naming	10.0 (3.9)	8.8 (3.1)	9.7 (2.8)	8.2 (3.9)	*F*(3,96) = 1.25, *p* = .30	0.04	0.326	–
Inhibition-inhibition	8.5 (3.7)	9.4 (3.3)	9.6 (2.1)	8.3 (3.8)	*F*(3,94) = 0.88, *p* = 0.45	0.03	0.236	–
Inhibition-switching	9.8 (3.6)	9.1 (2.8)	10.1 (2.7)	8.5 (4.1)	*F*(3,88) = 0.84, *p* = 0.48	0.03	0.226	–
Word-list interference	10.5 (3.3)	9.7 (3.6)	8.7 (3.9)	9.4 (4.0)	*F*(3,90) = 0.66, *p* = 0.58	0.02	0.184	–

In line with the NEPSY-2 manual, children < 7 years old did not complete Inhibition-Switching or Word List Interference

*p < 0.05. **p < 0.01. ***p <0 .001

^a^Controlled for age

^b^Controlled for sex and age

^c^Controlled for sex

### Diagnostic groups × Executive function (performance-based)

We found no significant differences between groups on NEPSY-2 subtests (see Table 2). All the group’s averaged scaled score means were between 8 and 12, i.e., within the expected level or normal range cf. NEPSY-2 manual [24].

### Irritability × Executive function (parent-report)

In the total sample, all BRIEF-2 Scales except working memory and task-monitor correlated significantly with irritability (see Table 3).

**Table 3:** Association between executive function and irritability in the total sample (*n* = 208)

	CBCL irritability		
	Correlation	Sig. (2-tailed)	β*
BRIEF-2			
Inhibition^a^	0.35	< 0.001	0.999
Self-monitor^a^	0.28	< 0.001	0.976
Cognitive flexibility ^a^	0.41	< 0.001	1.000
Emotion control^b^	0.76	< 0.001	1.000
Initiate^c^	0.21	0.005	0.830
Working memory^c^	0.10	0.185	0.279
Plan/organize^c^	0.25	< 0.001	0.937
Task-monitor^c^	0.09	0.234	0.235
Organization of materials^c^	0.18	0.017	0.701
NEPSY-2			
Cognitive flexibility	0.01	0.91	0.052
Inhibition-naming	0.00	0.98	0.050
Inhibition-inhibition	0.12	0.13	0.370
Inhibition-switching	0.04	0.60	0.085
Word-list interference	0.04	0.62	0.084

*With bias-correction and *p* = 0.05

^a^Partial-correlation controlling for sex and age

^b^Partial-correlation controlling for age

^c^Partial-correlation controlling for sex

### Irritability × Executive function (performance-based)

In the total sample, no significant correlations between irritability and NEPSY-2 subtests were found (see Table 3).

### Observed power

As evident from our results, each diagnostic group of interest consisted of 21–43 participants. Power analyses indicated that for BRIEF-2 measures the probability of a Type-II error ranged between 41.9 and 87.6%, and for NEPSY-2 measures between 67.4 and 88.1% (see Table 2 for β-values and Table 3 for β-values of our correlational analyses).

## Discussion

Our results show that children with DMDD display parent-reported emotional dyscontrol in daily life, both compared to validated norms and compared to children with ADHD. Furthermore, our results indicate that children with DMDD do not have problems with working memory in daily life unless they also have ADHD. Additionally, children with DMDD show clinical levels of cognitive inflexibility, which children with ADHD do not. No differences in EF are observed between children with DMDD or ODD.

Examining parent-reports of EF in daily life, we find that children with DMDD might have problems with emotion control and cognitive flexibility. This corresponds with existing research indicating emotion regulation difficulties [26] and reduced cognitive flexibility [15, 32] in these children. Importantly, our findings suggest that children with DMDD do not have problems with cognitive flexibility, inhibition, or working memory in test situations. These findings are in accordance with existing research showing no or ambiguous evidence of difficulties in these skills as measured by neuropsychological tasks in children with DMDD [31, 33]. Overall, by demonstrating problems with emotion control and cognitive flexibility in daily life, without executive difficulties in organized, relatively emotion-neutral situations, our results substantiate the hypothesis that children with DMDD might primarily have difficulties in EF related to emotion regulation, and not decontextualized problem-solving difficulties [14]. Instead, in daily life, they might get overwhelmed by feelings without accompanying regulatory capacities. In line with a developmental system perspective [41, 42], children with DMDD could have a maturational imbalance of coordination between affect and cognition.

Our study shows that children with DMDD or DMDD + ADHD have worse scores on emotional control as compared to children with ADHD-only. This suggests that deficits in the capacity to regulate subjective experience and expression of emotions, and the reduction of emotional arousal [43], is a unique, separate, and additional problem to ADHD that should be given specific attention in interventions.

By parent-report children with ADHD or DMDD + ADHD have significantly worse scores on working memory than those with DMDD or ODD suggesting that working memory difficulties could be attributed to ADHD specifically. By task-performance, however, children with ADHD or DMDD + ADHD do not have significantly worse scores on inhibition or working memory compared to children with DMDD, nor clinically elevated scores compared to validated norms. Although ADHD was included in this study for identifying unique DMDD mechanisms only, we notice that the literature is ambiguous and that some report that not all persons with ADHD have EF deficits as demonstrated by task-performance and that future research should refine neuropsychological tests for such assessments (e.g., [44, 45]).

In our study, children with DMDD or ODD show comparable scores on all measures. This could be interpreted as DMDD being a subtype of ODD. A challenge in making such statements based on parent-report, however, is that what seems behaviorally similar might be internally different. DMDD is linked to a heightened sensitivity to perceived threat or frustration [46] as well as excessive levels of arousal [30]. These are also characteristics of reactive aggression [47–49] referring to aggressive behavior displayed in response to a perceived threat or frustration [50]. Indeed, evidence indicates different subtypes of ODD; the ODD irritability type is also (i.e., like DMDD) associated with reactive aggression, whereas the defiant-behavior type is more strongly linked to proactive aggression [51, 52]. Proactive aggression refers to calculated aggressive behavior intended to achieve a desired outcome [50]. In line with theories and data distinguishing these types of aggression [47–49, 53, 54], the aggressive behavior of children with DMDD might therefore be less intentional as compared to the aggressive behavior of those with ODD (i.e., the defiant type). From a psychological perspective, observing the aggressive behavior of children with DMDD versus ODD in different socioemotional situations might give insights to such (u)intentional differences. Also their interpretations of these situations (e.g., hostile intent attribution) and their response evaluations (e.g., to what extent they approve their aggressive behavior) may support this assumption (see for example the interactive virtual-reality procedure in [54]).

In line with our predictions, we find that irritability is related to emotional dyscontrol and cognitive inflexibility in daily life. This further supports the hypothesis of higher levels of irritability being closely associated with emotion-related executive dysfunction, i.e., skills needed in situations that are motivationally significant [23]. It also corresponds with findings of irritability being positively associated with cognitive flexibility-related neural activity [17]. The causal relationship between emotional aspects of EF and irritability remains unanswered. Presumably, based on the developmental system perspective [41, 42], the irritable state reflects a tension between increased activity in body-neural networks related to emotions and attempts by the lateral prefrontal networks, i.e., the cognitive control system, to down-regulate the increased activity by for example providing convenient interpretations of the internal state. Accordingly, if regulation fails, tension releases in the form of anger outburst.

As with diagnoses, irritability is not associated with performance-based EF, including inhibition. Nevertheless, we did find a weak association between disinhibition and irritability by parent-report. Existing research is also ambiguous regarding the linkage between irritability and inhibition. One study demonstrates a weak or potentially nonexistent relationship between irritability and performance-based inhibition [18], whereas another study finds no significant associations between irritability and performance-based motor inhibition [55]. Notably, the latter study included children with ADHD only. Furthermore, a third study links higher levels of irritability to compensatory increases in prefrontal cortex during inhibition tasks [6].

Evidently, our study shows little correspondence between parent-rating and performance-based results. Recent literature indicates a strong discord between EF rating scales and performance-based tasks [56]. Measurements by task-performance are criticized for bearing little resemblance to real world situations in which EFs are used to guide behavior [56].

In our study, difficulties with emotion control and cognitive flexibility is found in children with higher levels of irritability and DMDD in measures reflecting daily functioning. No clear evidence of performance-based or parent-reported executive dysfunction of other EFs is demonstrated. This may suggests that children with DMDD or severe irritability have more problems in individually relevant day-to-day situations as compared to more emotionally neutral or structured environments. An essential opportunity for future research on DMDD and irritability is to clarify the relationship between situational content and the individual’s experience and expression of irritability and anger. This seems possible by children reporting their interpretations and intentions in socioemotional situations constructed for eliciting anger, including observation of their behavior in these situations [e.g., 54]. In treatment, intact EFs in structured situations could be understood as a meaningful resource by enabling exploration of more convenient understandings of their internal states and situations in which the child tends to get emotionally overwhelmed, including how they can react differently and thereby increasing their emotional control and cognitive flexibility.

### Strengths and limitations

A strength of our study is that all measurements were done as part of the children’s ordinary clinical assessment in child psychiatric services. This limit the burden (e.g., time spent on assessments) on the children and their families and is cost-effective for the clinics. A limitation is the relatively small diagnostic sample sizes indicative of reduced statistical power. These findings, especially the performance-based, therefore need replication in larger samples. As FSIQ was only measured if the healthcare professional found it necessary as part of the child’s clinical assessment, there might be a bias in terms of who received such assessment. One might expect those with lower functioning to have been IQ-assessed resulting in negatively skewed IQ means. Still, with DMDD being a new diagnosis in need of urgent attention and treatment innovations, our study brings knowledge on potential DMDD mechanisms.

In conclusion, our findings indicate that in daily life, children with higher levels of irritability or DMDD experience emotional dyscontrol and cognitive inflexibility. Our study also suggests that there are EF differences between children with DMDD and those with ADHD, but not between children with DMDD and those with ODD.

## Supplementary Information

Below is the link to the electronic supplementary material.

Supplementary file1 (DOCX 13 KB)

## Data Availability

The data that support the findings of this study are not publicly available due to them containing information that could compromise research participant privacy/consent.
