# Supplementary material for: Executive function in children with disruptive mood dysregulation disorder compared to attention-deficit/hyperactivity disorder and oppositional defiant disorder, and in children with different irritability levels
Source: Eur Child Adolesc Psychiatry. 2023 Jan 21;33(1):115–25. doi: 10.1007/s00787-023-02143-6 (PMC9867548; doi:10.1007/s00787-023-02143-6)
Supplement: Supplementary file 1 — Supplementary file1 (DOCX 13 KB) [file 787_2023_2143_MOESM1_ESM.docx]

Supporting Information I, The effect of COVID-19

Conducting data collection in times of COVID-19 where normal operating where occasionally closed or reduced, resulted in some participants not being able to complete NEPSY-2 (which require physical presence) and that the collection of some parent-reports were not followed up by the administrative clinicians due to sick leave. This does not change any of our presented data or results. We decided not to exclude participants that did not complete all measurements of the total study (NCT05049356 and REK2017/135) and followed the general principles from our recruitment procedure.

Supporting Information II, Sample size estimates

Pre data collection (cf. the project description), it was estimated that, based on current literature, approximately 60 of 200 children in this clinical sample would have DMDD, and that, based on existing research reporting statistically significant differences with the number of participants per group less than 20, that groups of 70 (DMDD) and 130 (non-DMDD) participants would be large enough to detect differences in test results in the present study. However, as it was decided as more clinically meaningful to compare children with DMDD to associated diagnoses of ADHD and ODD (instead of a pooled non-DMDD group), and that literature had found statistically significant differences with participant numbers less than 20, we assumed that groups of approximately 20 participants would be large enough to detect differences. Nevertheless, observed power (β) was calculated to investigate the probability of Type-II error/statistical power.
